# Health-Related Quality of Life for Patients with Post-Acute COVID-19 Syndrome: Identification of Symptom Clusters and Predictors of Long-Term Outcomes

**DOI:** 10.1007/s11606-024-08688-9

**Published:** 2024-02-29

**Authors:** Brittany Lapin, Yadi Li, Kristin Englund, Irene L. Katzan

**Affiliations:** 1 Center for Outcomes Research and Evaluation, Neurological Institute, Cleveland Clinic, Cleveland, OH USA; 2 Department of Quantitative Health Sciences, Lerner Research Institute, Cleveland Clinic, Cleveland, OH USA; 3 Department of Infectious Disease, Respiratory Institute, Cleveland Clinic, Cleveland, OH USA

## Abstract

**Background:**

Following COVID-19 infection, as many as a third of patients have long-term symptoms, known as post-acute sequelae (PASC). The mechanisms contributing to PASC remain largely unknown and, due to the heterogeneity of symptoms, treating PASC provides unique challenges.

**Objective:**

Our study sought to (1) identify clinical symptom profiles based on PROMIS Global Health (GH) items, (2) evaluate demographic and clinical differences across profiles, and (3) identify predictors of change in health-related quality of life (HRQL) over time.

**Design:**

This was an observational cohort study of patients with PASC who completed PROMIS-GH between 2/11/21 and 12/3/21 as part of routine care, with data extracted from the electronic health record.

**Participants:**

There were 1407 adult patients (mean age 49.6 ± 13.7, 73% female, 81% White race) with PASC seen in the recovery clinic between 2/11/21 and 12/3/21, with 1129 (80.2%) completing PROMIS-GH as routine care.

**Main Measures:**

HRQL was measured with PROMIS-GH at initial visit and after 12 months.

**Key Results:**

Latent profile analysis identified symptom classes based on five PROMIS-GH items (mental health, ability to carry out physical activities, pain, fatigue, and emotional problems). Four latent profiles were identified: (1) “Poor HRQL” (*n* = 346), (2) “Mixed HRQL: good mental/poor physical” (*n* = 232), (3) “Mixed HRQL: poor mental/good physical” (*n* = 324), and (4) “Good HRQL” (*n* = 227). Demographics and comorbidities varied significantly across profile with patients with more severe COVID-19 infection more likely to be in profiles 1 and 2. Overall, patients improved 2 T-score points on PROMIS-GH after 12 months, with differences by profile. Predictors of improved HRQL included profile, lower body mass index, and fewer COVID symptoms.

**Conclusions:**

Patients with PASC have distinct HRQL symptom profiles which were able to differentiate across COVID-19 severity and symptoms. Improvement over 12 months differed by profile. These profiles may be used to better understand the mechanisms behind PASC. Future research should evaluate their ability to guide treatment decisions to improve HRQL.

**Supplementary Information:**

The online version contains supplementary material available at 10.1007/s11606-024-08688-9.

## INTRODUCTION

Following COVID-19 infection, as many as a third to a half of patients are left with long-term symptoms, known as post-acute sequelae of SARS-CoV-2 infection (PASC). ^1–4^ PASC has been defined as a condition where symptoms continue for more than 12 weeks after infection with COVID-19, regardless of whether the initial infection resulted in symptoms. ^2^ Symptoms vary widely but most commonly include anxiety, ^5^ fatigue, cognitive problems, depression, anosmia, body aches, and an inability to regain pre-infection levels of health-related quality of life (HRQL). ^6^ PASC impairs activities of daily living ^5^ and disrupts work, home, and social life. ^7^ It is considered a multisystem and multiorgan disease affecting neurological, cardiac, and respiratory systems. ^3,8^ The mechanisms contributing to PASC remain largely unknown and, due to the heterogeneity of symptoms, treating PASC provides unique challenges. ^4^ Multidisciplinary approaches to treating patients with PASC, through post-COVID-19 clinics, have become the recommended treatment approach. ^9^

Patient-reported outcome measures (PROMs) have been utilized to understand patient symptoms and challenges when treating PASC. PROMIS Global Health (GH) is a measure of global HRQL and comprises 10 items which cover multiple domains affected with PASC including mental health, physical functioning, pain, fatigue, and emotional well-being. ^10^ Given the impact of PASC on multiple areas of life which are difficult to assess without patient report, questionnaires such as PROMIS-GH can be used to identify symptom constellations. Identifying distinct profiles of patients based on self-reported HRQL may enable greater understanding of the different mechanisms underlying PASC symptoms. It may also help guide individualized treatment and inform prognoses for recovery.

Our study sought to (1) identify clinical symptom profiles based on PROMIS-GH items, (2) evaluate demographic and clinical differences across profiles, (3) identify predictors of improvement in HRQL over time across profiles, and (4) evaluate how many patients returned to their pre-COVID level of HRQL in a subset of patients with historical PROMIS-GH data.

## METHODS

We conducted an observational cohort study of patients with PASC seen in a multidisciplinary COVID-19 recovery clinic. Patients were included in our study if they were ≥ 18 years, were seen in the reCOVer Clinic between 2/11/21 and 12/3/21, and completed PROMIS-GH as routine care.

COVID-19 clinical outcomes were extracted from the COVID-19 Cleveland Clinic Registry including hospitalization status, intensive care unit (ICU) admission, and symptoms. Severe initial COVID-19 was defined as illness requiring hospitalization or ICU stay. Demographics, select comorbidities, and PROMs were extracted from the electronic medical record (EMR).

The study was approved by Cleveland Clinic’s Institutional Review Board (#20–1331). Because the study consisted of analyses of pre-existing data, the requirement for patient informed consent was waived.

### reCOVer Clinic

The reCOVer Clinic at the Cleveland Clinic opened 2/10/21 to care for patients with persistent COVID-19-related symptoms. Patients diagnosed with PASC are referred to the reCOVer Clinic for a comprehensive evaluation and, from there, are referred to the appropriate specialty/specialties which have tailored care paths for patients with PASC.

At the initial reCOVer Clinic visit, symptoms were evaluated and defined for this study as symptoms that were self-reported as being present at initial COVID illness and not yet resolved by the reCOVer Clinic visit.

### Patient-Reported Outcome Measures

As part of routine care in the reCOVer Clinic, patients complete PROMs including PROMIS-GH v1.2, computerized adaptive test versions of PROMIS Sleep Disturbance, PROMIS Fatigue, and Neuro-QoL Cognitive Function, Generalized Anxiety Disorder (GAD)-2, Patient Health Questionnaire (PHQ)-2, and PTSD Checklist for DSM-5 (PCL-5).

PROMIS-GH is a 10-item measure of global health and includes a summary score for physical and mental global health. ^10^ PROMIS-GH, PROMIS Sleep Disturbance, PROMIS Fatigue, and Neuro-QoL Cognitive Function scores are standardized to a reference population on a T-scale with mean 50 and standard deviation (SD) 10 where higher scores indicate more of the domain being measured (i.e., higher scores on PROMIS-GH and Neuro-QoL Cognitive Function indicate better global health and cognitive function, respectively, whereas higher PROMIS Sleep Disturbance and Fatigue indicate more symptoms). These scales have been demonstrated as valid and reliable measures, with a change of 2.5–5 T-score points generally considered clinically meaningful. ^11–16^

### Pre-COVID Global Health and Long-Term Outcomes

Since 2015, PROMIS-GH has been completed across many institutes at Cleveland Clinic, including primary care. As a measure of pre-COVID global health, all PROMIS-GH measures collected between 1/1/2019 and 12/31/2019 were extracted for patients who were later seen in the reCOVer Clinic. For patients with multiple PROMIS-GH measures, the most recent measure was used for analysis.

Additionally, PROMIS-GH was extracted from the EMR for patients who completed it in another clinical department following their initial reCOVer Clinic visit. These follow-up measures were grouped into 3-, 6-, and 12-month follow-up based on time since the initial visit to the reCOVer Clinic.

### Statistical Analysis

#### Identification of Clinical Symptom Profiles

To identify symptom cluster profiles, latent profile analysis (LPA) was conducted using MPlus version 8.4. LPA is an analytic approach that uses model-based probabilities to group patients into similar symptom classes. ^20^ Initially, LPA identified symptom profiles based on all 10 PROMIS-GH items. Items 3 (physical health) and 9 (ability to carry out social activities) were removed from the analysis based on content overlap with similar items. An LPA model was initially constructed with a single symptom class and then successively built with an increasing number of symptom classes (i.e., the second model had two classes, the third model had three classes, etc.). The optimal number of symptom classes was determined by iteratively comparing the model with *k* classes with the model with (*k* − 1) classes using multiple fit indices (Supplemental Table 1). ^21,22^ Items 1 (general health), 2 (quality of life), and 5 (social satisfaction) did not differentiate the profiles so were removed from the LPA. Ultimately, five PROMIS-GH items were retained to create the profiles: items 4 (mental health), 6 (ability to carry out physical activities), 7 (pain), 8 (fatigue), and 10 (emotional problems). Upon selection of the model with the optimal number of symptom classes, patients were grouped into their most likely latent classes using estimated posterior membership probabilities.

#### Evaluation of Demographic and Clinical Differences Across Profiles

Patient characteristics were compared across the clinical symptom profiles using the chi-square test for categorical variables and ANOVA or the Kruskal–Wallis test for continuous variables, as appropriate.

#### Identification of Predictors of Improved HRQL Across Profiles

Change in PROMIS-GH at 3-, 6-, and 12-month follow-up was compared across clinical symptom profiles using ANOVA with Tukey’s post hoc test. Mixed-effects models were constructed to identify characteristics associated with improvement in PROMIS-GH physical and mental summary scores across time (from baseline at the reCOVer Clinic visit through 12-month follow-up). Patient was included as a random effect, and characteristics were included as fixed effects based on clinical relevance. To determine whether there were differential improvements in patients based on profile, a profile by time interaction effect was included in the models.

#### Subset Analysis of Return to Pre-COVID HRQL

A subset analysis was conducted for patients with pre-COVID PROMIS-GH and 12-month follow-up. Return to pre-COVID levels of global health was defined as those within 2.5 T-score points of their pre-COVID PROMIS-GH by 12-month follow-up. Characteristics associated with return to pre-COVID levels of global health were evaluated with the chi-square test for categorical variables and the *t*-test or Mann–Whitney *U* test for continuous variables, as appropriate.

Statistical analyses were conducted using SAS version 9.4 (SAS Institute Inc., Cary NC). All tests were two-sided and *p*-values less than 0.05 were considered statistically significant.

## RESULTS

There were 1407 unique patients seen for an initial consultation in the reCOVer Clinic, with 1129 patients completing PROMIS-GH and included in our study. The average age of the cohort was 49.6 (SD 13.7) years, 73.3% female and 81.4% White race (Table 1).

**Table 1:** Patient Characteristics, Comorbidities, COVID-19 Outcomes, and Symptoms by Latent Profiles

	Total *N* = 1129	Profile 1 Poor global health *N* = 346	Profile 2 Mixed: good mental/poor physical *N* = 232	Profile 3 Mixed: poor mental/good physical *N* = 324	Profile 4 good global health *N* = 227	*p*-value
Demographics						
Age, mean ± SD	49.6 ± 13.7	49.7 ± 13.3	52.5 ± 13.0	47.0 ± 13.5	50.2 ± 14.5	** < *0.001***^***a,2,4,6***^
Female, *n* (%)	827 (73.3)	278 (80.3)	165 (71.1)	233 (71.9)	151 (66.5)	***0.002***^***c,1,2,3***^
Race, *n* (%)						***0.004***^***c,2,4,5***^
White	919 (81.4)	268 (77.5)	176 (75.9)	284 (87.7)	191 (84.1)	
Black	144 (12.8)	52 (15.0)	37 (15.9)	27 (8.3)	28 (12.3)	
Other	66 (5.8)	26 (7.5)	19 (8.2)	13 (4.0)	8 (3.5)	
Hispanic, *n* (%)	41 (3.7)	10 (3.0)	12 (5.3)	14 (4.4)	5 (2.3)	0.30^c^
BMI (kg/m^2^), mean ± SD	31.6 ± 8.2	33.2 ± 8.8	33.3 ± 9.3	29.6 ± 6.9	30.5 ± 7.1	** < *0.001***^***a,2,3,4,5***^
Obese (30 + kg/m^2^), *n* (%)	516 (52.9)	186 (62.6)	113 (56.2)	120 (43.2)	97 (48.7)	** < *0.001***^***c,2,3,4***^
Comorbidities, *n* (%)						
Asthma	194 (17.2)	76 (22.0)	43 (18.5)	49 (15.1)	26 (11.5)	***0.007***^***c,2,3,5***^
COPD	28 (2.5)	10 (2.9)	8 (3.4)	8 (2.5)	2 (0.88)	0.32^c^
Coronary artery disease	36 (3.2)	12 (3.5)	13 (5.6)	8 (2.5)	3 (1.3)	0.056^c^
Diabetes	111 (9.8)	39 (11.3)	35 (15.1)	27 (8.3)	10 (4.4)	** < *0.001***^***c,3,4,5***^
Hypertension	276 (24.4)	91 (26.3)	73 (31.5)	67 (20.7)	45 (19.8)	***0.008***^***c,4,5***^
COVID outcomes, *n* (%)						
Hospitalization	303 (28.2)	117 (36.4)	81 (36.0)	55 (17.6)	50 (23.0)	** < *0.001***^***c,2,3,4,5***^
ICU stay	58 (5.4)	26 (8.1)	19 (8.5)	7 (2.2)	6 (2.8)	** < *0.001***^***c,2,3,4,5***^
Intubated	23 (2.1)	9 (2.8)	10 (4.4)	2 (0.63)	2 (0.91)	***0.009***^***d,4,5***^
COVID symptoms, *n* (%)						
Shortness of breath	758 (76.1)	247 (83.7)	170 (81.7)	212 (72.4)	129 (64.5)	** < *0.001***^***c,2,3,4,5***^
Cough	412 (40.2)	145 (47.5)	103 (47.9)	96 (32.1)	68 (32.9)	** < *0.001***^***c,2,3,4,5***^
Chest pain	512 (50.3)	195 (64.4)	108 (50.9)	128 (43.1)	81 (39.5)	** < *0.001***^***c,1,2,3,5***^
Palpitations	544 (54.4)	208 (67.3)	99 (50.0)	163 (56.0)	74 (36.6)	** < *0.001***^***c,1,2,3,5,6***^
Exertional intolerance	815 (82.8)	274 (91.9)	192 (92.3)	217 (77.0)	132 (67.3)	** < *0.001***^***c,2,3,4,5,6***^
Fatigue	912 (90.6)	294 (98.0)	194 (91.5)	266 (90.8)	158 (78.2)	** < *0.001***^***c,1,2,3,5,6***^
Dizziness	585 (58.4)	216 (72.0)	126 (61.2)	161 (54.9)	82 (40.4)	** < *0.001***^***c,1,2,3,5,6***^
Syncope	119 (11.9)	60 (19.6)	21 (10.2)	21 (7.3)	17 (8.5)	** < *0.001***^***c,1,2,3***^
Fever	41 (4.0)	20 (6.4)	8 (3.8)	9 (3.0)	4 (1.9)	***0.049***^***c,2,3***^
Joint pain/body aches	628 (62.9)	237 (79.0)	133 (64.3)	167 (57.6)	91 (45.0)	** < *0.001***^***c,1,2,3,5,6***^
Altered taste/smell	454 (45.2)	153 (51.5)	83 (39.2)	144 (49.3)	74 (36.5)	***0.001***^***c,1,3,4,6***^
Exhaustion/prolonged fatigue	823 (82.9)	277 (93.6)	181 (87.0)	238 (83.5)	127 (62.3)	** < *0.001***^***c,1,2,3,5,6***^
Lack of concentration/brain fog	758 (75.9)	269 (90.0)	145 (68.7)	235 (81.3)	109 (54.5)	** < *0.001***^***c,1,2,3,4,5,6***^
Memory deficits	696 (70.4)	258 (86.6)	129 (62.3)	212 (73.6)	97 (49.5)	** < *0.001***^***c,1,2,3,4,5,6***^
Diarrhea/nausea	395 (39.3)	162 (53.6)	74 (35.6)	118 (39.6)	41 (20.8)	** < *0.001***^***c,1,2,3,5,6***^
Headaches	653 (64.3)	238 (78.0)	125 (59.8)	195 (65.4)	95 (46.8)	** < *0.001***^***c,1,2,3,5,6***^
Difficulty sleeping	771 (78.1)	261 (87.0)	157 (76.6)	223 (76.9)	130 (67.7)	** < *0.001***^***c,1,2,3,5,6***^
Orthopnea/edema	272 (28.5)	113 (39.2)	63 (32.0)	59 (20.8)	37 (19.7)	** < *0.001***^***c,2,3,4,5***^
*Total symptoms, median (Q1, Q3)*	*10.0 (6.0, 12.0)*	*12.0 (9.0, 14.0)*	*10.0 (7.0, 12.0)*	*9.0 (6.0, 12.0)*	*7.0 (3.0, 10.0)*	** < *0.001***^***b,1,2,3,5,6***^
PROMs at reCOVer Clinic visit, mean ± SD						
PCL-5*	20.0 ± 16.7	32.3 ± 17.4	11.4 ± 9.4	22.2 ± 14.7	7.4 ± 8.3	** < *0.001***^***a,1,2,3,4,6***^
GAD-2*	2.2 ± 2.0	3.5 ± 1.9	1.1 ± 1.5	2.8 ± 1.9	0.69 ± 1.1	** < *0.001***^***a,1,2,3,4,6***^
PHQ-2*	2.0 ± 1.9	3.4 ± 1.8	1.2 ± 1.3	2.0 ± 1.7	0.63 ± 1.07	** < *0.001***^***a,1,2,3,4,5,6***^
Neuro-QoL Cognitive Function	39.2 ± 9.9	32.7 ± 7.3	41.7 ± 8.5	38.3 ± 8.0	47.6 ± 9.9	** < *0.001***^***a,1,2,3,4,5,6***^
PROMIS Fatigue*	62.9 ± 9.2	68.9 ± 7.2	63.2 ± 7.2	61.5 ± 7.6	54.6 ± 9.3	** < *0.001***^***a,1,2,3,5,6***^
PROMIS Sleep Disturbance*	57.7 ± 8.2	62.1 ± 8.2	56.3 ± 7.3	57.2 ± 7.5	53.1 ± 7.0	** < *0.001***^***a,1,2,3,5,6***^

Data not available for all subjects, missing values: Hispanic = 33; BMI = 154; hospitalization = 54; ICU = 49; intubated = 48; SOB = 133; cough = 103; chest pain = 112; palpitations = 129; exertional intolerance = 145; fatigue = 122; dizziness = 127; syncope = 131; fever = 93; joint pain = 130; taste/smell = 125; exhaustion = 136; brain fog = 130; memory = 140; diarrhea = 124; headaches = 114; sleeping = 142; orthopnea = 173; total symptoms = 67; PCL = 616; GAD2 = 380; PHQ2 = 159; Neuro-QoL Cognition = 432; PROMIS Fatigue = 315; PROMIS Sleep = 344. Common comorbidities were chosen to provide information on patients’ general health, with asthma and chronic obstructive pulmonary disease (COPD) included due to symptom overlap with PASC

COVID symptoms are those that were present during initial infection and had not resolved by the reCOVer Clinic visit

^*^Higher scores indicate worse symptoms. The PHQ-2 comprises the first 2 items of the Patient Questionaire-9 (PHQ-9), and is a validated tool that inquiries about the degree to which an individual has experienced depressed mood and anhedonia over the past 2 weeks. ^17^ The GAD-2 is a 2-item questionnaire related to the frequency of anxiety symptoms over the previous 15 days. ^18^The PCL-5 is a 20-item measure that screens individuals for PTSD. Scores > 31 are indicative of probable PTSD^19^

*p*-values: a = ANOVA, b = Kruskal–Wallis test, c = Pearson’s chi-square test, d = Fisher’s exact test; bold and italicized values are statistically significant, p<0.05

Significant pairwise comparisons based on Tukey’s post hoc test: 1 = profile 1 vs 2 significantly different; 2 = profile 1 vs 3 significantly different; 3 = profile 1 vs 4 significantly different; 4 = profile 2 vs 3 significantly different; 5 = profile 2 vs 4 significantly different; 6 = profile 3 vs 4 significantly different, *p* < 0.05

### Identification of Clinical Symptom Profiles

**Figure 1:**
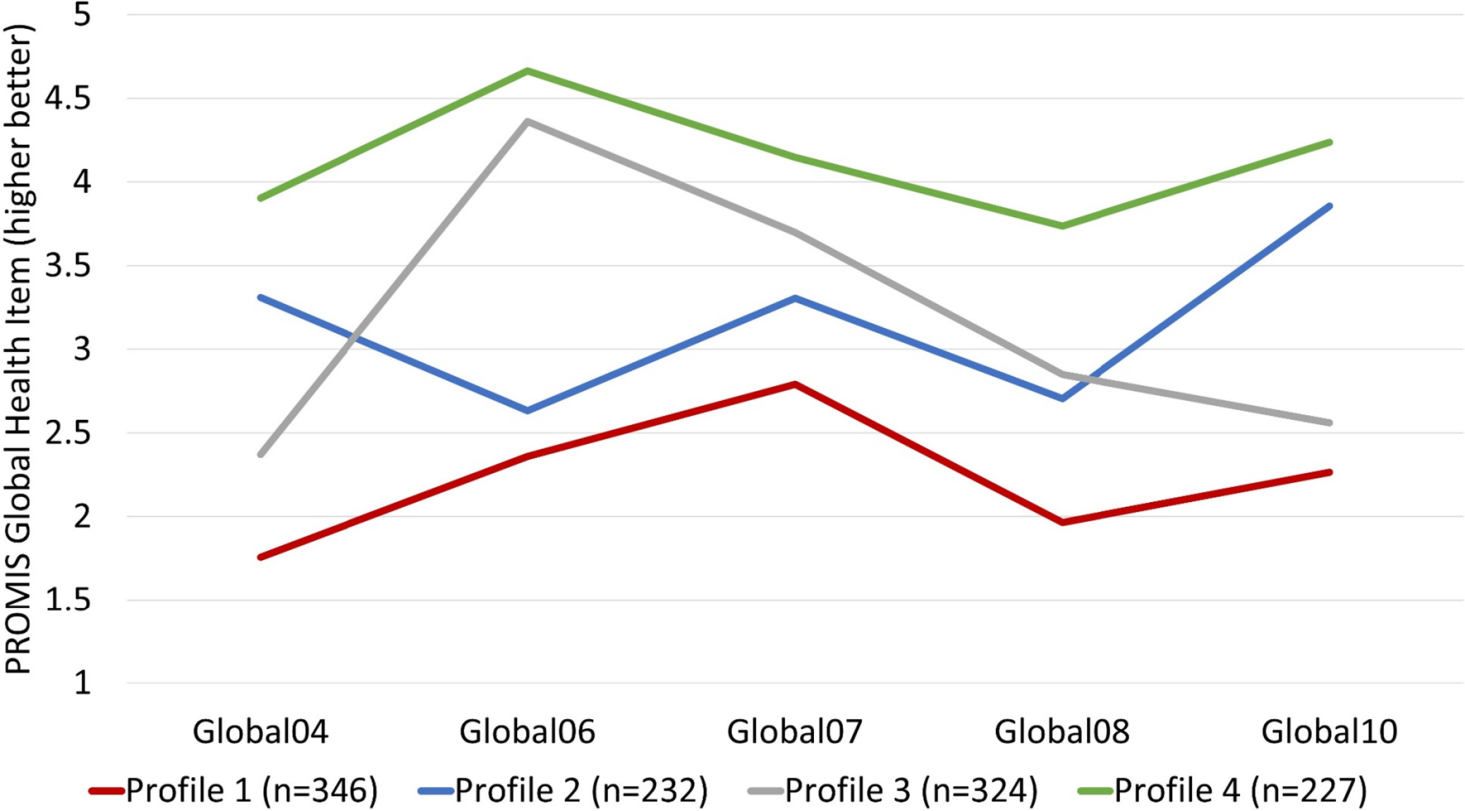
Latent profiles in patients with PASC, *n* = 1129. Footnote: Global04 = mental health including mood and ability to think; Global06 = ability to carry out every physical activities; Global07 = pain; Global08 = fatigue; Global10 = emotional problems such as feeling anxious and depressed. The model classified patients well, with average posterior probabilities of 0.89 (SD = 0.15), 0.86 (0.14), 0.86 (0.16), and 0.90 (0.15) in profiles 1–4, respectively.

LPA identified four latent profiles as having the best fit for the data: (1) “Poor global health” (*n* = 346), (2) “Mixed: good mental/poor physical” (*n* = 232), (3) “Mixed: poor mental/good physical” (*n* = 324), and (4) “Good global health” (*n* = 227) (Supplemental Table 1; Fig. 1).

### Evaluation of Demographic and Clinical Differences Across Profiles

Demographics, comorbidities, COVID outcomes and symptoms, and PROMs varied significantly across profile (Table 1). There were significantly more women with poor global health compared to good global health (80.3% vs 66.5%). Patients in profiles 1 and 2 (poor global physical) were more likely to be of Black race and have more comorbidities and more severe COVID-19 infection. Patients in profile 3 (“Mixed: poor mental/good physical”) were the youngest, were more likely to be of White race, had fewer comorbidities, and had less severe initial COVID-19. Similarly, patients in profile 4 with good global health had fewer comorbidities and less severe initial COVID-19.

PROMs at the time of the initial reCOVer Clinic visit also differed by profile (Table 1). Across all PROMs, scores were best for those in profile 4 and worst in profile 1. For profiles 2 and 3, PROMs that captured concepts more related to physical health, including fatigue, were worse for those in profile 2 (with poor global physical), while PROMs capturing concepts more related to mental health (PCL-5, anxiety, depression, cognitive function) were worse for those in profile 3 (with poor global mental).

### Identification of Predictors of Improved HRQL Across Profiles

Improvement in PROMIS-GH from the reCOVer Clinic visit occurred incrementally from 3 to 12 months (Table 2; Supplemental Fig. 1). Overall, patients improved 2.1 (SD 7.6) T-score points on global mental health and 1.9 (SD 6.1) T-score points on global physical health by 12 months. There were significant differences by profile, with patients in profile 1 (poor global health) improving the most and those in profile 4 (good global health) remaining stable.

**Table 2:** PROMIS Global Health over Time by Latent Profile

	Total	Profile 1 Poor global health	Profile 2 Mixed: good mental/poor physical	Profile 3 Mixed: poor mental/good physical	Profile 4 Good global health	*p*-value
reCOVer Clinic PROMIS-GH, mean ± SD	*N* = 1129	*N* = 346	*N* = 232	*N* = 324	*N* = 227	
Global mental health	41.5 ± 9.2	32.8 ± 5.9	45.2 ± 5.6	40.4 ± 5.4	52.7 ± 6.2	** < *0.001***^***a,1,2,3,4,5,6***^
Global physical health	39.8 ± 6.9	33.3 ± 4.1	37.1 ± 3.8	43.1 ± 3.9	47.9 ± 4.6	** < *0.001***^***a,1,2,3,4,5,6***^
Months between COVID diagnosis and reCOVer Clinic visit, median [Q1, Q3]	5.8 [3.5, 8.7]	5.5 [3.3, 8.5]	5.3 [3.2, 8.5]	6.1 [4.1, 8.9]	5.9 [3.8, 8.7]	0.081^b^
Follow-up PROMIS-GH, mean ± SD						
3 months, *n*	*N* = 727	*N* = 230	*N* = 159	*N* = 206	*N* = 132	
Global mental health	41.7 ± 9.2	34.6 ± 6.9	45.0 ± 7.2	40.8 ± 6.6	51.6 ± 7.1	** < *0.001***^***a,1,2,3,4,5,6***^
Change	0.58 ± 5.8*	2.0 ± 6.1*	− 0.35 ± 5.2	0.83 ± 5.2*	− 1.2 ± 6.3*	** < *0.001***^***a,1,3,6***^
Global physical health	40.4 ± 6.7	35.2 ± 5.0	39.0 ± 5.4	42.9 ± 5.1	47.0 ± 5.2	** < *0.001***^***a,1,2,3,4,5,6***^
Change	0.80 ± 4.5*	2.0 ± 4.3*	1.8 ± 4.6*	− 0.19 ± 3.7	− 0.95 ± 4.7*	** < *0.001***^***a,2,3,4,5***^
6 months, *n*	*N* = 512	*N* = 163	*N* = 106	*N* = 151	*N* = 92	
Global mental health	43.1 ± 9.5	36.4 ± 7.1	44.7 ± 8.6	43.8 ± 7.8	52.1 ± 8.4	** < *0.001***^***a,1,2,3,5,6***^
Change	1.9 ± 7.3*	4.0 ± 6.7*	− 0.73 ± 7.2	3.1 ± 7.2*	− 0.85 ± 7.1	** < *0.001***^***a,1,3,4,6***^
Global physical health	41.2 ± 6.8	37.0 ± 5.4	39.1 ± 6.0	43.7 ± 5.4	46.9 ± 5.9	** < *0.001***^***a,1,2,3,4,5,6***^
Change	1.7 ± 5.5*	3.7 ± 5.1*	2.2 ± 5.8*	0.68 ± 5.2	− 0.79 ± 5.2	** < *0.001***^***a,2,3,5***^
12 months, *n*	*N* = 847	*N* = 262	*N* = 165	*N* = 248	*N* = 172	
Global mental health	43.6 ± 9.4	37.3 ± 7.9	45.6 ± 8.2	43.3 ± 7.4	51.6 ± 8.4	** < *0.001***^***a,1,2,3,4,5,6***^
Change	2.1 ± 7.6*	4.5 ± 7.7*	0.38 ± 7.1	2.9 ± 6.8*	− 1.03 ± 7.7	** < *0.001***^***a,1,3,4,6***^
Global physical health	41.8 ± 6.9	37.3 ± 5.8	40.4 ± 5.9	44.2 ± 5.6	46.8 ± 6.4	** < *0.001***^***a,1,2,3,4,5,6***^
Change	1.9 ± 6.1*	3.6 ± 6.0*	3.4 ± 5.9*	0.98 ± 5.4*	− 0.74 ± 6.1	** < *0.001***^***a,2,3,4,5,6***^

Change compared to reCOVer Clinic scores at baseline; positive change indicates improvement. *Significant change based on paired *t*-test, *p* < 0.05. Three-, 6-, and 12-month follow-up defined as 15–135, 136–240, and > 240 days, respectively, following baseline score

*p*-values: a = ANOVA, b = Kruskal–Wallis test, c = Pearson’s chi-square test, d = Fisher’s exact test

Significant pairwise comparisons based on Tukey’s post hoc test: 1 = profile 1 vs 2 significantly different; 2 = profile 1 vs 3 significantly different; 3 = profile 1 vs 4 significantly different; 4 = profile 2 vs 3 significantly different; 5 = profile 2 vs 4 significantly different; 6 = profile 3 vs 4 significantly different, *p* < 0.05

After adjustment, in a multivariable mixed-effects model where predictors for inclusion were determined a priori, PROMIS-GH scores improved over time for all groups (Table 3). A differential effect was found with significant interaction effects between profile and time, which demonstrated greater improvements in global mental health for those in profiles 1 and 3 (with poor global mental), and greater improvements in global physical health for those in profiles 1 and 2 (with poor physical health) (Supplemental Fig. 2).

**Table 3:** Multivariable Mixed-Effects Models for Improvement in Global Health Over Time (*N* = 1129)

	PROMIS global mental health		PROMIS global physical health	
	Estimate (SE)	*p*-value	Estimate (SE)	*p*-value
Age (per decade)	0.511 (0.160)	***0.001***	− 0.026 (0.114)	0.816
Female	0.189 (0.454)	0.677	0.293 (0.323)	0.365
Obese (BMI ≥ 30 kg/m^2^)	− 0.506 (0.412)	0.219	− 1.124 (0.293)	** < *0.001***
COPD	− 1.024 (1.192)	0.391	− 1.824 (0.848)	***0.032***
Diabetes	− 0.087 (0.704)	0.902	0.050 (0.500)	0.921
Hypertension	− 0.044 (0.506)	0.931	− 0.333 (0.360)	0.355
Hospitalization for COVID	0.383 (0.447)	0.392	− 0.223 (0.318)	0.482
Total number of COVID symptoms	− 0.228 (0.052)	** < *0.001***	− 0.214 (0.037)	** < *0.001***
Months between COVID diagnosis and reCOVer Clinic visit	0.003 (0.053)	0.953	− 0.039 (0.038)	0.306
Time point (reference = baseline)				
3 months	0.667 (0.279)	***0.017***	0.935 (0.213)	** < *0.001***
6 months	1.829 (0.318)	** < *0.001***	1.905 (0.243)	** < *0.001***
12 months	2.240 (0.262)	** < *0.001***	2.302 (0.200)	** < *0.001***
Profile (reference = profile 1)				
Profile 2	9.368 (0.576)	** < *0.001***	2.615 (0.409)	** < *0.001***
Profile 3	5.930 (0.537)	** < *0.001***	7.267 (0.382)	** < *0.001***
Profile 4	15.600 (0.611)	** < *0.001***	10.511 (0.435)	** < *0.001***

Significant time by profile interaction graphed in Supplemental Figs. 2a and 2b

Characteristics associated with improved global mental health from the time of the reCOVer Clinic visit through 12 months included increased age and fewer COVID symptoms. Being male and having lower body mass index were also predictors at the bivariate level, but were no longer associated after adjustment (Supplemental Table 2). Except for age, which was not associated with change, characteristics associated with improved global physical health were like those for global mental health. Worsening over time for global physical health occurred for patients with prior comorbidities, as well as those with worse initial COVID outcomes.

### Subset Analysis of Return to Pre-COVID HRQL

For the subset of patients with pre-COVID PROMIS-GH (*n* = 497), questionnaires were completed a median of 3.4 (IQR 1.4–7.4) months prior to initial COVID-19 infection. Overall, only 31.5% and 36.9% of patients returned to their pre-COVID levels of global mental and physical health after 12 months, respectively, with no difference by profile (Table 4).

**Table 4:** Patients Return to Pre-COVID Level of HRQL at 12-Month Follow-Up

	Total *N* = 497	Profile 1 Poor HRQOL *N* = 140	Profile 2 Mixed: good mental/poor physical *N* = 99	Profile 3 Mixed: poor mental/good physical *N* = 152	Profile 4 Good HRQOL *N* = 106	*p*-value
Historical PROMIS-GH						
Months before initial COVID diagnosis, median [q1, q3]	3.4 [1.4, 7.4]	3.0 [1.3, 6.4]	2.7 [1.1, 7.5]	3.5 [1.3, 7.7]	3.9 [1.9, 9.3]	0.14^b^
Global mental health, mean ± SD	45.7 ± 9.3	39.2 ± 7.8	47.7 ± 8.3	45.0 ± 7.2	53.6 ± 8.4	** < *0.001***^***a,1,2,3,4,5,6***^
Global physical health, mean ± SD	43.9 ± 6.6	39.0 ± 5.8	42.6 ± 5.6	45.9 ± 4.9	48.9 ± 5.7	** < *0.001***^***a,1,2,3,4,5,6***^
PROMIS global mental health						0.97^c^
Returned to pre-COVID level, *n* (%)	156 (31.5)	46 (32.9)	30 (30.3)	48 (31.6)	32 (30.5)	
Did not return to pre-COVID level, *n* (%)	340 (68.6)	94 (67.1)	69 (69.7)	104 (68.4)	73 (69.5)	
PROMIS global physical health						0.90^c^
Returned to pre-COVID level, *n* (%)	183 (36.9)	51 (36.4)	37 (37.4)	53 (35.1)	42 (39.6)	
Did not return to pre-COVID level, *n* (%)	313 (63.1)	89 (63.6)	62 (62.6)	98 (64.9)	64 (60.4)	

Subset of patients with PROMIS Global Health scores pre-COVID (in 2019), at reCOVer Clinic visit and 12-month follow-up

Return to pre-COVID level is defined as 12-month follow-up score within ± 2.5 T-score points of pre-COVID score

*p*-values: a = ANOVA, b = Kruskal–Wallis test, c = Pearson’s chi-square test, d = Fisher’s exact test

Significant pairwise comparisons based on Tukey’s post hoc test: 1 = profile 1 vs 2 significantly different; 2 = profile 1 vs 3 significantly different; 3 = profile 1 vs 4 significantly different; 4 = profile 2 vs 3 significantly different; 5 = profile 2 vs 4 significantly different; 6 = profile 3 vs 4 significantly different, *p* < 0.05

Trajectories of improvement over time by profile were similar in the subset of patients with pre-COVID PROMIS-GH as compared to the full cohort (Supplemental Fig. 3). Apart from less COVID-related fever and syncope, no characteristics were associated with patients returning to their pre-COVID levels of global health (Supplemental Table 3).

## DISCUSSION

Patients with PASC have distinct global health profiles which were able to differentiate across initial COVID-19 severity and symptoms. Our study identified four distinct clinical symptom profiles based on mental health, physical function, fatigue, pain, and emotional health. Patient profiles included (1) those with overall poor global health, (2) those with good mental health but poor physical health, (3) those with but poor mental health but good physical health, and (4) those with overall good global health. Overall, global health, PROMIS Fatigue and Sleep Disturbance, Neuro-QoL Cognitive Function, and depression scores in our study were meaningfully worse than the general population, however were similar to those reported in other studies of PASC patients. ^23,24^

Seventy-three percent of the patients in our study were women, and significantly, more women were in the poor global health profile. Other studies have found women are more affected by long-COVID than males. ^25,26^ Women may have a different COVID-19 immune response compared to men, and more persistent post-COVID symptoms. ^26^ There were other demographic and clinical differences across profiles; patients in profiles with poor physical health (profiles 1 and 2) were more likely to be Black, had more persistent COVID symptoms and more comorbid conditions, and were more likely to have had a severe initial COVID-19 infection. Prior studies have found that patients from racial and ethnic minority groups have worse outcomes following COVID-19 infection and have significantly more PASC symptoms. ^27,28^ In our study, Black patients had more comorbidities and worse initial COVID illness as evidenced by higher rates of ICU stays and intubations compared to White patients. After controlling for comorbidities and COVID severity, associations between race and profile were eliminated, suggesting it is comorbidities and COVID severity, rather than race, which influence profile membership.

Our study found overall slight improvement in self-reported global health over 12 months of follow-up. However, the degree of improvement varied by profile and clinical characteristics. Patients in profiles with poor physical health scores (profiles 1 and 2) had the greatest improvement in physical scores over the following 12-month period, which averaged 3.6 T-score points in patients in profile 1 (poor global health) and 3.4 points in those in profile 2 (poor physical/good mental health), exceeding minimal clinically important differences. Analogously, patients in profiles with poor mental health scores (profiles 1 and 3) had the greatest improvement in mental health scores over time, with patients in profile 1 (poor global health) improving 4.5 T-score points, and patients in profile 3 (poor mental/good physical) improving 2.9 points. This degree of average change is modest but likely noticeable by patients. In contrast, patients in profiles with good physical and/or mental health scores did not have clinically relevant improvement in scores over time.

In our study, more unresolved COVID-19 symptoms were significantly associated with lower self-reported physical and mental health and less improvement over time. This is consistent with findings from other studies. ^29,30^ A longitudinal study of patients with COVID-19 infection found worse HRQL at 12 months for patients with more than one initial COVID symptom. ^25^ Garg et al. found symptoms during acute illness did not relate to reduced PROMIS-GH scores for patients seen in a PASC clinic, but patients with multiple symptoms at 3 months post-infection did have worse scores. ^30^ Similarly, another study of patients presenting to a post-COVID clinic found worse HRQL scores for patients with more remaining symptoms. ^31^ In our study, across all profiles, more COVID symptoms and more comorbidities were associated with worsening HRQL over time.

Interestingly, demographics were largely unassociated with changes in HRQL over time. Despite women and patients of Black race being more likely to be in profiles with worse physical HRQL, these characteristics were unassociated with improvement or worsening over time. A longitudinal study of patients followed up for 18 months following COVID infection found that women and those older than 65 years of age were more likely to experience declines in HRQL. ^29^ That study had a high percentage of patients who were hospitalized for COVID and HRQL may differ across demographics based on severity of disease. Other studies have shown older age was associated with greater rates of PASC and more PASC symptoms. ^32,33^ In our study, older age was significantly related to improvement in mental health over time. As older age is in general associated with better self-reported mental health, ^34^ it is unknown how much our findings can be attributed to recovery from PASC.

Our study is unique in that a large subset of patients (*n* = 497) had pre-COVID global health surveys available with which to aid in interpretation of post-COVID scores. Patients in profiles with poor PASC physical health scores (profiles 1 and 2) had the worst pre-COVID physical health scores. Despite having poor pre-COVID physical health, these patients exhibited the largest decline in physical health when they developed PASC compared to pre-COVID levels. Similarly, PASC patients in profiles with poor mental health scores (profiles 1 and 3) had the worst pre-COVID mental health scores. Even so, these patients experienced the largest decline in mental health scores compared to their pre-COVID levels. These findings—that patients with poor pre-COVID physical and/or mental health scores have a greater decline in these scores, respectively, with PASC—suggest these patients may be particularly susceptible to the negative impact of PASC. Fortunately, these patients also experienced greater improvements in their initially poor physical and mental health scores such that approximately one-third of patients in each of the four profiles returned to pre-COVID scores by 12 months of follow-up. Interestingly, there were no characteristics associated with returning to pre-COVID levels of HRQL, aside from initial pre-COVID HRQL.

The goal of PASC clinics is to provide individualized patient-centered care. Our identified clinical symptom profiles provide insights on expected recovery. Based on our findings, patients in profiles with poor mental and/or physical health are more likely to experience greater improvement in self-reported health at 12 months, whereas patients with good global health are likely to remain stable over 1 year. Despite modest improvements in self-reported health for patients with initially poor scores, only one-third of patients in any of the profiles returned to their pre-COVID health. These trajectories can be used to help estimate the degree of anticipated improvement based on self-reported health at presentation to recovery clinics.

Our study had many strengths including a representative sample of patients with PASC receiving care at a multidisciplinary treatment center, and PROMs completed as standard care, which increases generalizability of our findings. Unlike most studies which rely on recall of pre-COVID health and comparison to published norms, our study provides actual pre-COVID HRQL measures collected in routine care through a large health system. Despite these strengths, there are some limitations that deserve mention. This was a retrospective study, and PROMs included pre-COVID and during follow-up are based on who returned for care. Patients who may be doing well may not follow-up, and patients with historically poorer HRQL may have been more likely to have a pre-COVID survey. Patients who completed surveys and were included in our study were more likely of White race; however, other characteristics largely did not differ. We also did not have information on how patients were treated following their reCOVer Clinic visit, and future studies should evaluate subsequent referrals and the ability of the clinical symptom profiles to inform referrals to improve HRQL and symptoms in patients with PASC.

In conclusion, our study identified unique profiles based on PROMIS-GH that were able to discriminate across patients with varying symptoms and initial COVID severities. While the degree of improvement in patient-reported health over 12 months of follow-up varied by patient profile, most patients in each profile did not return to their pre-COVID levels of HRQL. As the healthcare needs for patients with PASC continue to increase, our study provides distinct symptom profiles to help clinicians develop targeted treatments.

### Supplementary Information

Below is the link to the electronic supplementary material.

Supplementary file1 (PDF 507 KB)

## References

[1] Yoo SM, Liu TC, Motwani Y (2022). Factors associated with post-acute sequelae of SARS-CoV-2 (PASC) after diagnosis of symptomatic COVID-19 in the inpatient and outpatient setting in a diverse cohort. J Gen Intern Med..

[2] Augustin M, Schommers P, Stecher M (2021). Post-COVID syndrome in non-hospitalised patients with COVID-19: a longitudinal prospective cohort study. Lancet Reg Health Eur..

[3] Groff D, Sun A, Ssentongo AE (2021). Short-term and long-term rates of postacute sequelae of SARS-CoV-2 infection: a systematic review. JAMA Netw Open..

[4] Nalbandian A, Sehgal K, Gupta A (2021). Post-acute COVID-19 syndrome. Nat Med..

[5] Tabacof L, Tosto-Mancuso J, Wood J (2022). Post-acute COVID-19 syndrome negatively impacts physical function, cognitive function, health-related quality of life, and participation. Am J Phys Med Rehabil..

[6] Bahmer T, Borzikowsky C, Lieb W (2022). Severity, predictors and clinical correlates of Post-COVID syndrome (PCS) in Germany: a prospective, multi-centre, population-based cohort study. EClinicalMedicine..

[7] Havervall S, Rosell A, Phillipson M (2021). Symptoms and functional impairment assessed 8 months after mild COVID-19 among health care workers. JAMA..

[8] Barbato C, Di Certo MG, Gabanella F (2021). Staying tuned for post-COVID-19 syndrome: looking for new research to sniff out. Eur Rev Med Pharmacol Sci..

[9] **Vehar S, Boushra M, Ntiamoah P, Biehl M.** Update to post-acute sequelae of SARS-CoV-2 infection: caring for the ‘long-haulers’. Cleve Clin J Med*.* 2021 May 3;88(5):267-272. 10.3949/ccjm.88a.21010 33941600

[10] Hays RD, Bjorner JB, Revicki DA, Spritzer KL, Cella D (2009). Development of physical and mental health summary scores from the patient-reported outcomes measurement information system (PROMIS) global items. Qual Life Res..

[11] Buysse DJ, Yu L, Moul DE (2010). Development and validation of patient-reported outcome measures for sleep disturbance and sleep-related impairments. Sleep..

[12] Norman GR, Sloan JA, Wyrwich KW (2003). Interpretation of changes in health-related quality of life: the remarkable universality of half a standard deviation. Med Care..

[13] Cella D, Lai JS, Jensen SE (2016). PROMIS Fatigue Item Bank had clinical validity across diverse chronic conditions. J Clin Epidemiol..

[14] Cella D, Lai JS, Nowinski CJ (2012). Neuro-QOL: brief measures of health-related quality of life for clinical research in neurology. Neurology..

[15] Yost KJ, Eton DT, Garcia SF, Cella D (2011). Minimally important differences were estimated for six Patient-Reported Outcomes Measurement Information System-Cancer scales in advanced-stage cancer patients. J Clin Epidemiol..

[16] Meaningful Change for PROMIS®. https://www.healthmeasures.net/score-and-interpret/interpret-scores/promis/meaningful-change. Accessed 02/10/2021.

[17] Kroenke K, Spitzer RL, Williams JB (2003). The Patient Health Questionnaire-2: validity of a two-item depression screener. Med Care..

[18] Spitzer RL, Kroenke K, Williams JB, Lowe B (2006). A brief measure for assessing generalized anxiety disorder: the GAD-7. Arch Intern Med..

[19] Blevins CA, Weathers FW, Davis MT, Witte TK, Domino JL (2015). The Posttraumatic Stress Disorder Checklist for DSM-5 (PCL-5): development and initial psychometric evaluation. J Trauma Stress..

[20] Collins LM, Lanza ST (2010). Latent class and latent transition analysis: with applications in the social behavioral, and health sciences.

[21] **Lo Y, Mendell NR, D.B. R.** Testing the number of components in a normal mixture. Biometrika*.* 2001;88:767–778.

[22] McLachlan GJ, Peel D (2000). Finite mixture models.

[23] **Ryan M, Liang H, Wilson E, et al.** Quantifying the neuropsychiatric symptoms in post-acute sequelae of COVID-19 (PASC) using the NIH Toolbox ® and PROMIS. NeuroImmune Pharm Ther*.* 2023 Jun 20;2(2):95-101. 10.1515/nipt-2022-0010 PMC10373798 37502462

[24] **Perez Giraldo GS, Ali ST, Kang AK, et al.** Neurologic manifestations of long COVID differ based on acute COVID-19 severity. Ann Neurol*.* 2023 Jul;94(1):146-159. 10.1002/ana.26649 PMC10724021 36966460

[25] Seessle J, Waterboer T, Hippchen T (2022). Persistent symptoms in adult patients 1 year after coronavirus disease 2019 (COVID-19): a prospective cohort study. Clin Infect Dis..

[26] Takahashi T, Ellingson MK, Wong P (2020). Sex differences in immune responses that underlie COVID-19 disease outcomes. Nature..

[27] Webb Hooper M, Napoles AM, Perez-Stable EJ (2020). COVID-19 and racial/ethnic disparities. JAMA..

[28] Khullar D, Zhang Y, Zang C (2023). Racial/ethnic disparities in post-acute sequelae of SARS-CoV-2 infection in New York: an EHR-based cohort study from the RECOVER Program. J Gen Intern Med..

[29] Tanguay P, Decary S, Lemaire-Paquette S (2023). Trajectories of health-related quality of life and their predictors in adult COVID-19 survivors: a longitudinal analysis of the Biobanque Quebecoise de la COVID-19 (BQC-19). Qual Life Res..

[30] Garg A, Subramain M, Barlow PB (2023). Patient experiences with a tertiary care post-COVID-19 clinic. J Patient Exp..

[31] Lemhofer C, Sturm C, Loudovici-Krug D (2023). Quality of life and ability to work of patients with Post-COVID syndrome in relation to the number of existing symptoms and the duration since infection up to 12 months: a cross-sectional study. Qual Life Res..

[32] Zang C, Zhang Y, Xu J (2023). Data-driven analysis to understand long COVID using electronic health records from the RECOVER initiative. Nat Commun..

[33] Sudre CH, Murray B, Varsavsky T (2021). Attributes and predictors of long COVID. Nat Med..

[34] Thomas ML, Kaufmann CN, Palmer BW (2016). Paradoxical trend for improvement in mental health with aging: a community-based study of 1,546 adults aged 21–100 years. J Clin Psychiatry..

