# Supplementary material for: Health-Related Quality of Life for Patients with Post-Acute COVID-19 Syndrome: Identification of Symptom Clusters and Predictors of Long-Term Outcomes
Source: J Gen Intern Med. 2024 Feb 29;39(8):1301–9. doi: 10.1007/s11606-024-08688-9 (PMC11169186; doi:10.1007/s11606-024-08688-9)
Supplement: Supplementary file 1 — Supplementary file1 (PDF 507 KB) [file 11606_2024_8688_MOESM1_ESM.pdf]

SUPPLEMENTAL MATERIAL

Supplemental Table 1. Goodness-of-Fit Criteria for Solutions Specifying 2-6 Patient Symptom Profiles

**Classes**	**2**	**3**	**4**	**5**	**6**
AIC	15532.7	15300.8	15021.4	14957.6	14845.2
BIC	15613.2	15411.4	15162.2	15128.6	15046.3
Sample-size adjusted BIC	15562.3	15341.6	15073.3	15020.6	14919.3
Smallest profile proportion	46%	23%	20%	11%	11%
LMR-LRT	<0.001	0.003	<0.001	0.057	0.001
VLMR-LRT	<0.001	0.003	<0.001	0.060	0.001
BLRT	<0.001	<0.001	<0.001	<0.001	<0.001

AIC=Akaike information criterion; BIC=Bayesian information criterion; VLMR=Vuong-Lo-Mendell-Rubin Likelihood Ratio Test p-value; LMR=Lo-Mendell-Rubin Adjusted Likelihood Ratio Test p-value; BLRT = Bootstrapped Likelihood Ratio Test

Supplemental Figure 1a. Change in PROMIS Global Mental Health over Time, n=1129

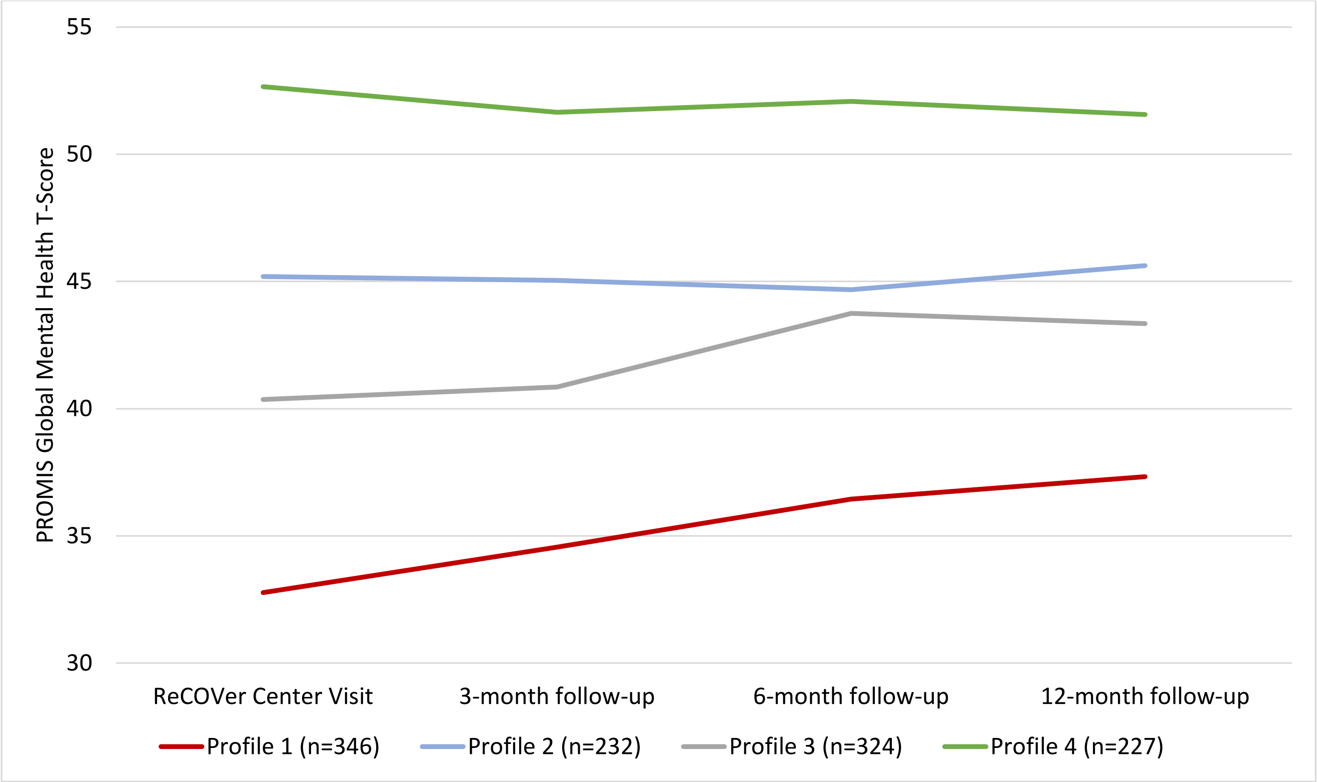

Supplemental Figure 1b. Change in PROMIS Global Physical Health over Time, n=1129

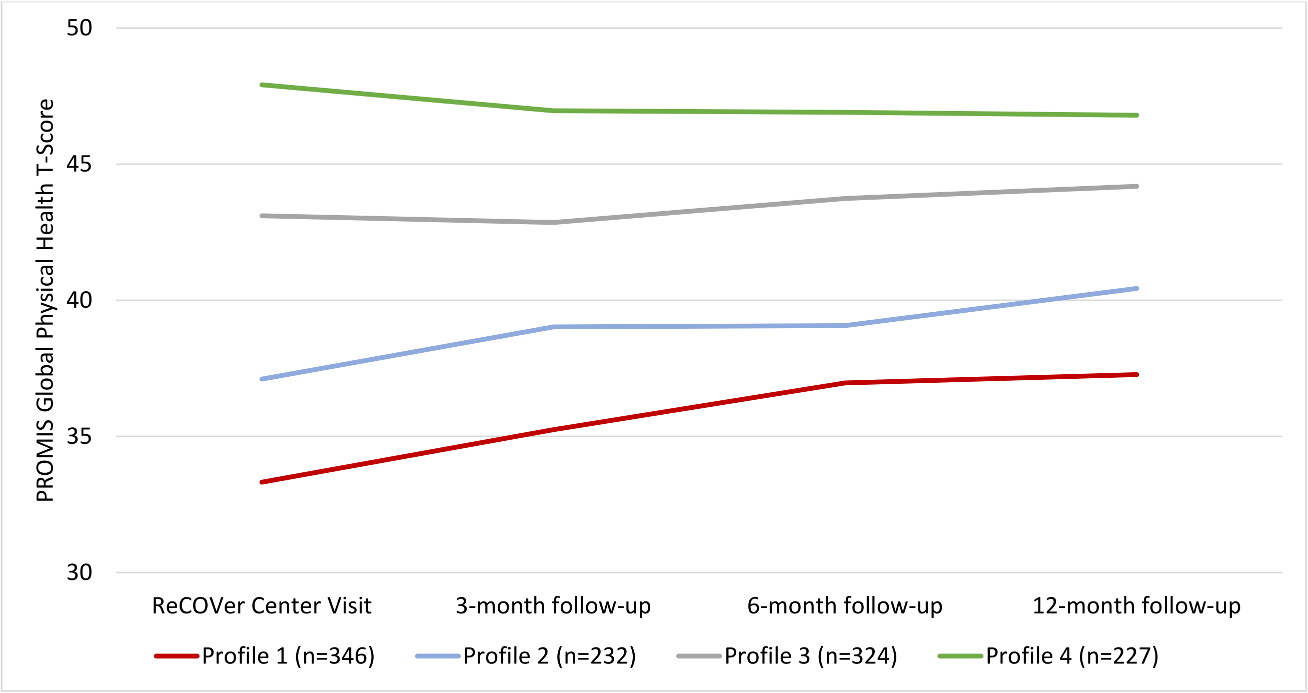

Supplemental Table 2. Bivariate mixed-effects models for Change in PROMIS Global Mental and Physical Health over time (baseline, 3-month, 6-month, 12-month)

	PROMIS Global Mental Health		PROMIS Global Physical Health	
	**Estimate (SE)**	**P-value**	**Estimate (SE)**	**P-value**
**Demographics**				
Age	0.060 (0.018)	***0.001***	-0.018 (0.014)	0.192
Female	-1.927 (0.570)	***0.001***	-1.059 (0.418)	***0.011***
Race				
White	-reference-		-reference-	
Black	-0.340 (0.762)	0.656	-0.771 (0.557)	0.167
Other	-2.120 (1.088)	0.052	-1.287 (0.797)	0.106
Hispanic	-1.763 (1.347)	0.191	-1.487 (0.985)	0.131
Body Mass Index (kg/m^2)	-0.131 (0.033)	***<0.001***	-0.196 (0.024)	***<0.001***
Obese (BMI ≥30 kg/m^2)	-1.791 (0.544)	***0.001***	-2.476 (0.393)	***<0.001***
**Comorbidities**				
Asthma	-0.724 (0.668)	0.279	-1.350 (0.487)	***0.006***
COPD	-1.975 (1.619)	0.223	-3.298 (1.181)	***0.005***
Coronary Artery Disease	-0.177 (1.422)	0.901	-1.615 (1.038)	0.120
Diabetes	-0.427 (0.846)	0.614	-1.572 (0.617)	***0.011***
Hypertension	-0.135 (0.587)	0.818	-1.489 (0.427)	***0.001***
**COVID Outcomes**				
Hospitalization	-0.667 (0.573)	0.245	-2.159 (0.412)	***<0.001***
ICU Stay	-1.644 (1.145)	0.151	-3.655 (0.830)	***<0.001***
Intubated	0.033 (1.791)	0.985	-3.392 (1.306)	***0.010***
**COVID Symptoms**				
Shortness of Breath	-2.602 (0.619)	***<0.001***	-3.699 (0.445)	***<0.001***

	PROMIS Global Mental Health		PROMIS Global Physical Health	
	**Estimate (SE)**	**P-value**	**Estimate (SE)**	**P-value**
Cough	-1.385 (0.531)	***0.009***	-2.162 (0.388)	***<0.001***
Chest Pain	-2.742 (0.518)	***<0.001***	-2.746 (0.379)	***<0.001***
Palpitations	-4.331 (0.520)	***<0.001***	-2.869 (0.384)	***<0.001***
Exertional Intolerance	-3.932 (0.700)	***<0.001***	-5.392 (0.498)	***<0.001***
Fatigue	-6.452 (0.887)	***<0.001***	-5.917 (0.650)	***<0.001***
Dizziness	-3.741 (0.526)	***<0.001***	-2.997 (0.387)	***<0.001***
Syncope	-3.540 (0.816)	***<0.001***	-3.153 (0.596)	***<0.001***
Fever	-2.809 (1.329)	***0.035***	-3.556 (0.975)	***<0.001***
Joint Pain/Body Aches	-4.000 (0.539)	***<0.001***	-3.700 (0.390)	***<0.001***
Altered Taste/Smell	-1.833 (0.525)	***0.001***	-0.387 (0.390)	0.321
Exhaustion/Prolonged Fatigue	-5.703 (0.694)	***<0.001***	-5.204 (0.501)	***<0.001***
Lack of Concentration/Brain Fog	-5.779 (0.597)	***<0.001***	-3.424 (0.449)	***<0.001***
Memory Deficits	-5.044 (0.564)	***<0.001***	-3.203 (0.422)	***<0.001***
Diarrhea/Nausea	-4.448 (0.522)	***<0.001***	-3.402 (0.384)	***<0.001***
Headaches	-3.603 (0.539)	***<0.001***	-2.415 (0.399)	***<0.001***
Difficulty Sleeping	-4.001 (0.637)	***<0.001***	-2.760 (0.470)	***<0.001***
Orthopnea/Edema	-2.587 (0.597)	***<0.001***	-3.256 (0.428)	***<0.001***
Total Symptoms	*-0.518 (0.054)*	***<0.001***	*-0.401 (0.039)*	***<0.001***

Independent variables in each model include time point (ReCOVer Center visit (baseline), 3-month, 6-month and 12-month) and one covariate. Subject random effect is included.

In separate models, profile group and interaction between covariate and profile group were added. Interactions with $p<0.05$ on omnibus test of interaction were graphed for interpretation. There were no clinically meaningful interaction effects. Positive estimates indicate improvement while negative estimates indicate worsening.

Supplemental Figure 2a. Interaction between profile group and time point, for PROMIS Global Mental Health (p<0.001)

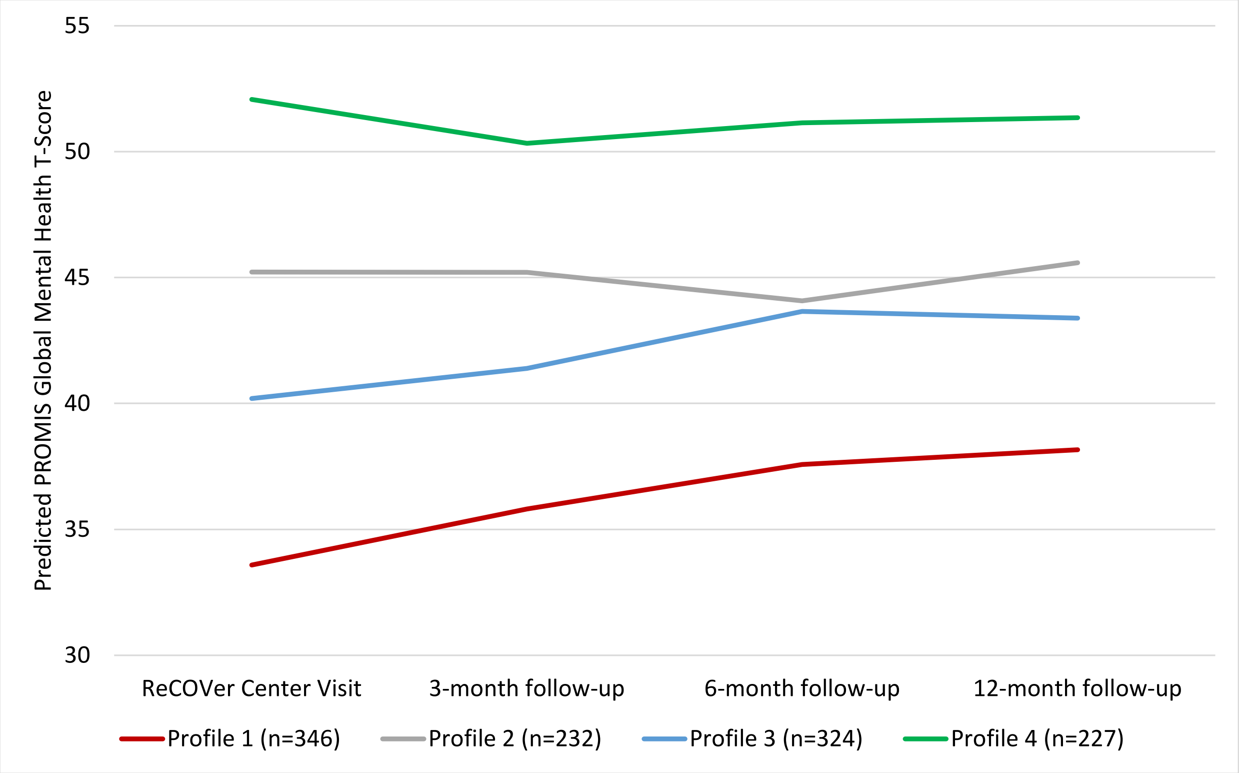

Based on mixed effects models presented in Table 3. Fit computed at mean age (50.39), mean number of COVID symptoms (9.59), mean time between COVID positive test and ReCOVer center visit (6.14 months), male, BMI<30, no hospitalization, no COPD, diabetes or hypertension.

Supplemental Figure 2b. Interaction between profile group and time point, for PROMIS Global Physical Health (p<0.001)

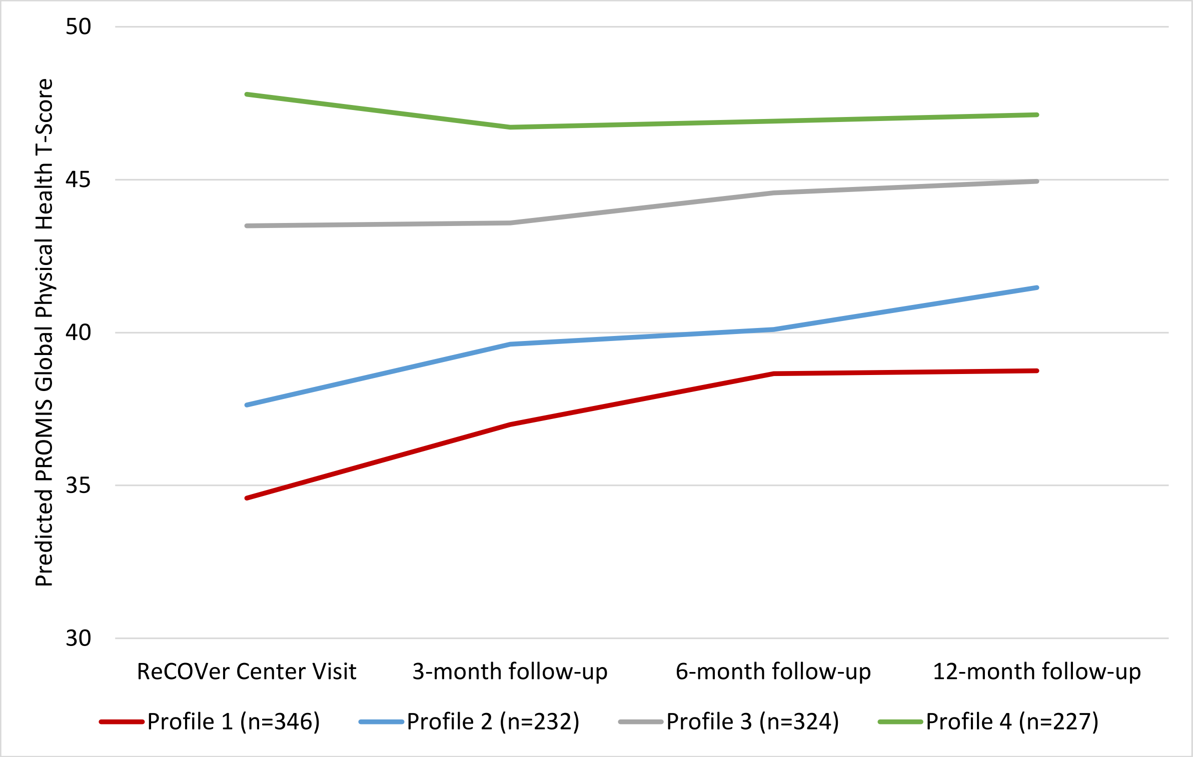

Based on mixed effects models presented in Table 3. Fit computed at mean age (50.39), mean number of COVID symptoms (9.59), mean time between COVID positive test and ReCOVer center visit (6.14 months), male, BMI<30, no hospitalization, no COPD, diabetes or hypertension.

Supplemental Figure 3a. Change in PROMIS Global Mental Health over Time, n=497

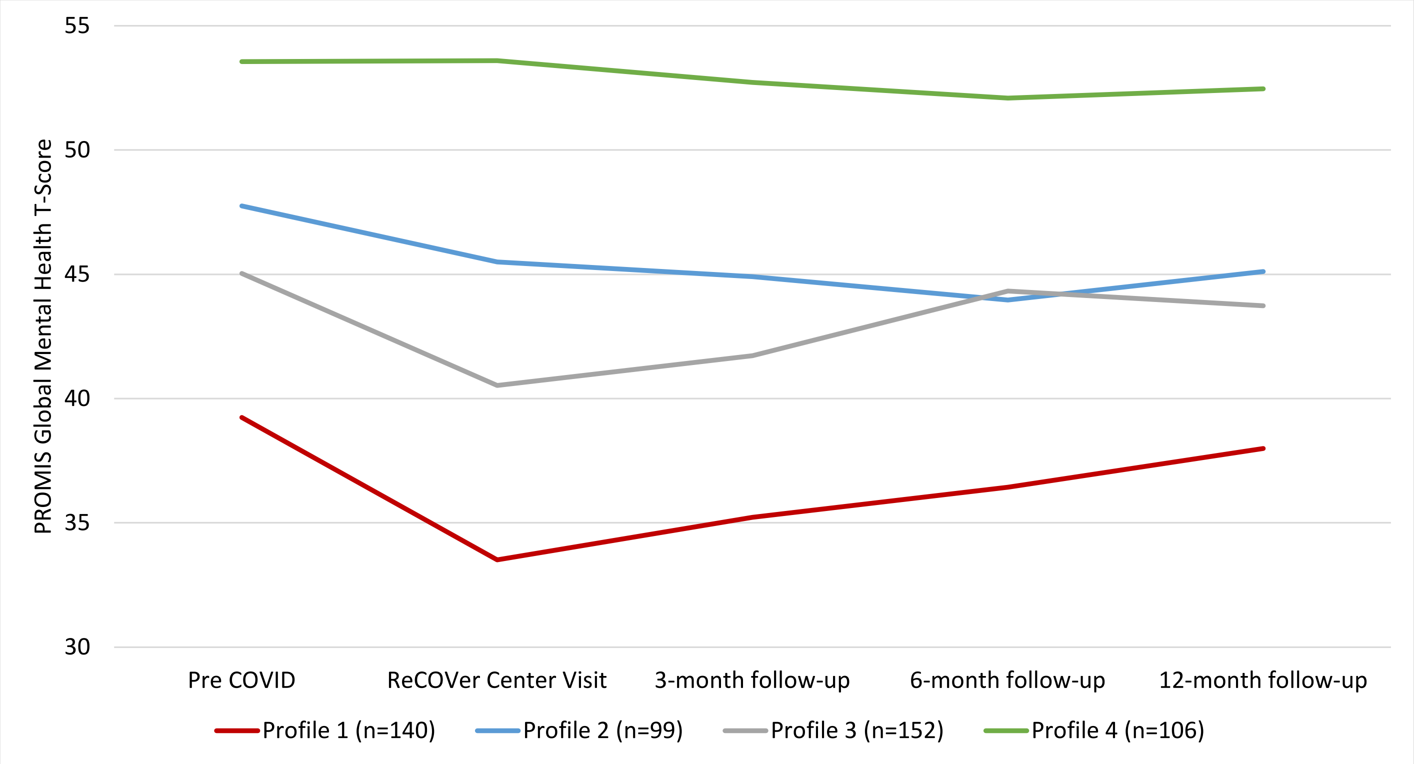

Subset of patients with PROMIS Global Health scores pre-COVID (in 2019), at ReCOVer Center visit, and 12-month follow-up

Supplemental Figure 3b. Change in PROMIS Global Physical Health over Time, n=497

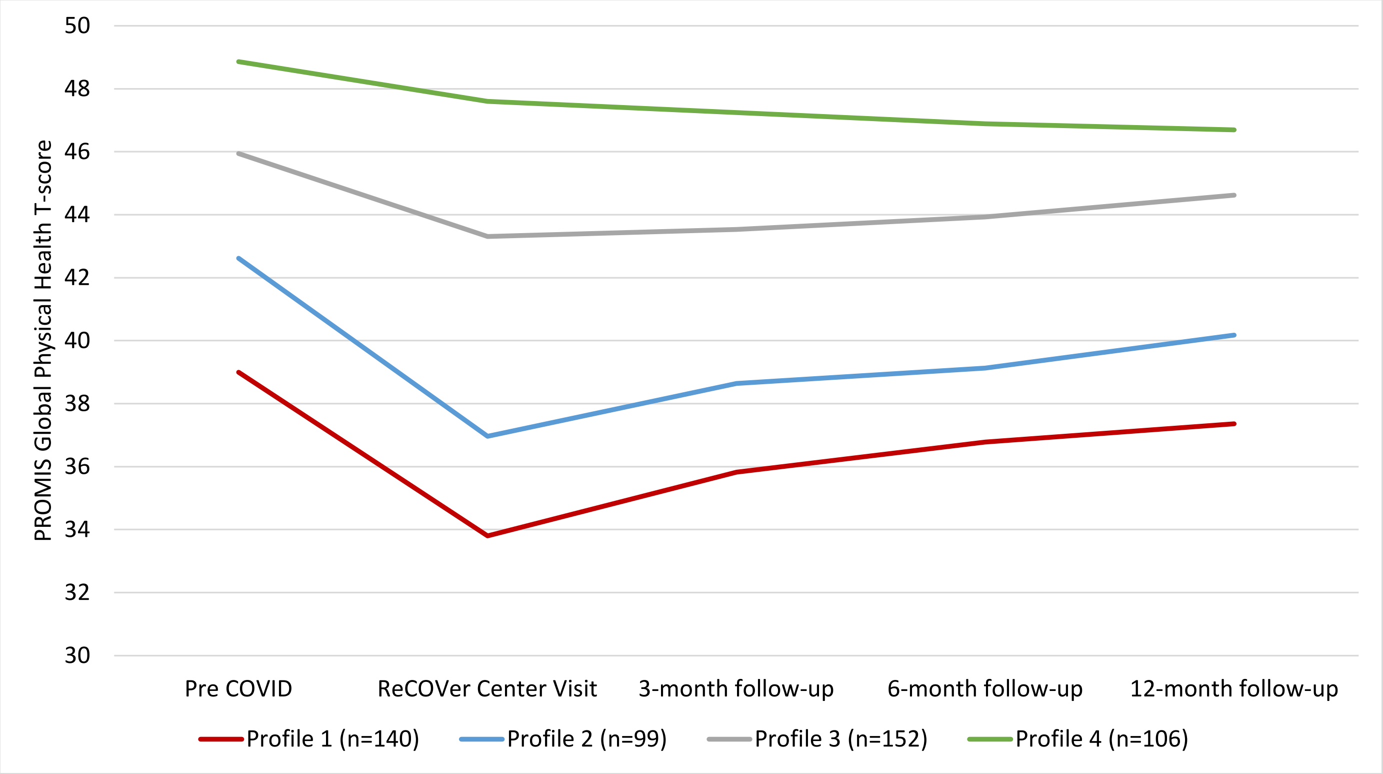

Subset of patients with PROMIS Global Health scores pre-COVID (in 2019), at ReCOVer Center visit, and 12-month follow-up

Supplemental Table 34. Patient Characteristics, Comorbidities, COVID Outcomes and Symptoms, stratified by return to pre-COVID level of PROMIS Global Mental and Physical Health

		Global Mental Health			Global Physical Health		
	**Total (N=496)**	**Did not return to pre-COVID level (N=340)**	**Returned to pre-COVID level (N=156)**	**P-value**	**Did not return to pre-COVID level (N=313)**	**Returned to pre-COVID level (N=183)**	**P-value**
**Demographics**							
Age, mean ± SD	51.5 ± 13.4	50.9 ± 13.8	52.8 ± 12.3	0.12[a1]	51.3 ± 13.4	52.0 ± 13.4	0.58[a]
Female, n (%)	377 (76.0)	257 (75.6)	120 (76.9)	0.75[c]	237 (75.7)	140 (76.5)	0.84[c]
Race, n (%)				0.92[c]			0.95[c]
White	406 (81.9)	278 (81.8)	128 (82.1)		257 (82.1)	149 (81.4)	
Black	65 (13.1)	44 (12.9)	21 (13.5)		41 (13.1)	24 (13.1)	
Other	25 (5.0)	18 (5.3)	7 (4.5)		15 (4.8)	10 (5.5)	
Hispanic, n (%)	17 (3.4)	13 (3.8)	4 (2.6)	0.47[c]	11 (3.5)	6 (3.3)	0.89[c]
BMI (kg/m^2), mean ± SD	32.6 ± 8.5	32.5 ± 8.1	33.0 ± 9.3	0.56[a2]	32.8 ± 8.6	32.3 ± 8.4	0.53[a]
Obese (BMI ≥30 kg/m^2), n (%)	255 (57.4)	170 (57.2)	85 (57.8)	0.91[c]	158 (56.6)	96 (58.2)	0.75[c]
**Comorbidities, n (%)**							
Asthma	109 (22.0)	79 (23.2)	30 (19.2)	0.32[c]	73 (23.3)	36 (19.7)	0.34[c]
COPD	13 (2.6)	11 (3.2)	2 (1.3)	0.36[d]	11 (3.5)	2 (1.09)	0.15[d]
Coronary Artery Disease	18 (3.6)	12 (3.5)	6 (3.8)	0.86[c]	12 (3.8)	6 (3.3)	0.75[c]
Diabetes	58 (11.7)	40 (11.8)	18 (11.5)	0.94[c]	41 (13.1)	17 (9.3)	0.20[c]
Hypertension	135 (27.2)	94 (27.6)	41 (26.3)	0.75[c]	92 (29.4)	44 (24.0)	0.20[c]
**COVID Outcomes, n (%)**							
Hospitalization	147 (30.7)	97 (29.9)	50 (32.3)	0.61[c]	90 (29.9)	57 (32.0)	0.63[c]
ICU stay	23 (4.8)	15 (4.6)	8 (5.2)	0.75[c]	13 (4.3)	10 (5.6)	0.50[c]
Intubated	10 (2.1)	6 (1.8)	4 (2.6)	0.73[d]	7 (2.3)	3 (1.7)	0.75[d]
**COVID Symptoms, n (%)**							

Shortness of Breath	351 (78.9)	243 (79.9)	108 (76.6)	0.42[c]	220 (79.1)	130 (77.8)	0.75[c]
Cough	195 (42.7)	137 (43.9)	58 (40.0)	0.43[c]	123 (43.2)	73 (42.4)	0.88[c]
Chest Pain	226 (50.2)	161 (52.4)	65 (45.5)	0.17[c]	138 (49.6)	87 (50.6)	0.85[c]
Palpitations	247 (54.6)	175 (56.5)	72 (50.7)	0.25[c]	156 (55.3)	91 (53.5)	0.71[c]
Exertional Intolerance	372 (84.5)	255 (85.0)	117 (83.6)	0.70[c]	236 (85.5)	135 (82.3)	0.37[c]
Fatigue	410 (90.5)	281 (90.4)	129 (90.8)	0.87[c]	257 (90.2)	153 (91.1)	0.75[c]
Dizziness	267 (59.3)	189 (62.0)	78 (53.8)	0.099[c]	171 (61.1)	96 (56.5)	0.34[c]
Syncope	49 (11.1)	43 (14.2)	6 (4.3)	***0.002[c]***	33 (12.1)	16 (9.5)	0.39[c]
Fever	15 (3.3)	14 (4.5)	1 (0.69)	***0.045[d]***	10 (3.5)	5 (2.9)	0.72[c]
Joint Pain/Body Aches	295 (66.3)	201 (66.6)	94 (65.7)	0.86[c]	192 (68.6)	103 (62.4)	0.19[c]
Altered Taste/Smell	205 (45.9)	147 (47.7)	58 (41.7)	0.24[c]	134 (47.5)	72 (43.6)	0.43[c]
Exhaustion/Prolonged Fatigue	375 (83.1)	256 (82.8)	119 (83.8)	0.80[c]	241 (84.9)	134 (80.2)	0.21[c]
Lack of Concentration/Brain Fog	346 (76.2)	235 (76.1)	111 (76.6)	0.91[c]	220 (76.1)	125 (75.8)	0.93[c]
Memory Deficits	324 (72.8)	225 (74.8)	99 (68.8)	0.18[c]	213 (75.5)	110 (67.5)	0.067[c]
Diarrhea/Nausea	189 (42.9)	136 (45.2)	53 (37.9)	0.15[c]	121 (44.0)	69 (41.6)	0.62[c]
Headaches	302 (66.1)	208 (66.2)	94 (65.7)	0.92[c]	189 (66.1)	114 (66.7)	0.90[c]
Difficulty Sleeping	342 (78.8)	232 (78.4)	110 (79.7)	0.75[c]	210 (77.8)	132 (80.5)	0.50[c]
Orthopnea/Edema	123 (29.1)	87 (29.8)	36 (27.5)	0.63[c]	83 (31.1)	40 (25.6)	0.23[c]
Total Symptoms	*10.0 [7.0, 12.5]*	*10.0 [6.0, 13.0]*	*10.0 [7.0, 12.0]*	*0.21[b]*	*10.0 [7.0, 13.0]*	*10.0 [6.0, 12.0]*	*0.68[b]*

Data not available for all subjects. Missing values: BMI = 52; Hospitalization = 17; ICU = 15; Intubated = 13; SOB = 51; Cough = 39; Chest Pain = 46; Palpitations = 44; Exertional Intolerance = 56; Fatigue = 43; Dizziness = 46; Syncope = 54; Fever = 37; Joint Pain = 51; Taste/Smell = 49; Exhaustion = 45; Brain Fog = 42; Memory = 51; Diarrhea = 55; Headaches = 39; Sleeping = 62; Orthopnea = 73.

p-values: a1=t-test, a2=Satterthwaite t-test, b=Wilcoxon Rank Sum test, c=Pearson's chi-square test, d=Fisher's Exact test.
